# Syntaxin-4 and SNAP23 are involved in neutrophil degranulation, but not in the release of mitochondrial DNA during NET formation

**DOI:** 10.3389/fimmu.2023.1272699

**Published:** 2023-10-09

**Authors:** Lea Gigon, Timothée Fettrelet, Marta Miholic, Kenneth R. McLeish, Shida Yousefi, Darko Stojkov, Hans-Uwe Simon

**Affiliations:** ^1^ Institute of Pharmacology, University of Bern, Bern, Switzerland; ^2^ Faculty of Pharmacy, University of Ljubljana, Ljubljana, Slovenia; ^3^ Department of Medicine, University of Louisville School of Medicine, Louisville, KY, United States; ^4^ Institute of Biochemistry, Brandenburg Medical School, Neuruppin, Germany

**Keywords:** degranulation, EET, eosinophils, mitochondrial DNA, NET, neutrophils, SNAP23, syntaxin-4

## Abstract

Neutrophils are a specialized subset of white blood cells, which have the ability to store pre-formed mediators in their cytoplasmic granules. Neutrophils are well-known effector cells involved in host protection against pathogens through diverse mechanisms such as phagocytosis, degranulation, extracellular traps, and oxidative burst. In this study, we provide evidence highlighting the significance of the SNARE proteins syntaxin-4 and synaptosomal-associated protein (SNAP) 23 in the release of azurophilic granules, specific granules, and the production of reactive oxygen species in human neutrophils. In contrast, the specific blockade of either syntaxin-4 or SNAP23 did not prevent the release of mitochondrial dsDNA in the process of neutrophil extracellular trap (NET) formation. These findings imply that degranulation and the release of mitochondrial dsDNA involve at least partially distinct molecular pathways in neutrophils.

## 1 Introduction

Neutrophils are bone marrow-derived white blood cells (WBCs) and belong to the granulocytes – a heterogeneous subtype of immune cells characterized by the prevalence of cytoplasmic granules, that also comprises basophils, mast cells, and eosinophils (1). Neutrophils play a crucial role as effector cells and are able to capture and eliminate invading microorganisms through diverse mechanisms such as phagocytosis, exocytosis of granule content, formation of neutrophil extracellular traps (NETs), and production of reactive oxygen species (ROS) (2, 3).

During the process of neutrophil differentiation, distinct types of granules are sequentially formed, allowing the storage of specific preformed mediators, including azurophilic (primary) granules, specific (secondary) granules, gelatinase (tertiary) granules, and secretory vesicles (SVs) (4, 5). A crucial step in neutrophil degranulation is the fusion of the granules with the plasma membrane, a process that is mediated by soluble N-ethylmaleimide-sensitive factor activating protein receptor (SNARE) proteins (4, 5). Notably, human neutrophils have been found to express several SNARE isoforms (6). Among them, syntaxin-4, and synaptosomal-associated protein (SNAP) 23 are predominantly located at the plasma and neutrophil granule membranes, where they appear to have a regulatory function in exocytosis (4, 7).

NETs are extracellular structures defined by the association of cytotoxic granule proteins with a double-stranded DNA (dsDNA) scaffold, which contribute to the antimicrobial activity of neutrophils (8). It is worth mentioning that various other immune cells have been reported to form extracellular traps such as eosinophils (9–11), mast cells (12), and basophils (13), to name a few. Although the source of the DNA scaffold and the requirement for neutrophil death in NET formation are still subjects of ongoing scientific dispute (14, 15), compelling evidence suggests that viable neutrophils have the ability to release mitochondrial DNA (mtDNA) without affecting their longevity (16, 17). The mechanism of NET formation was shown to rely on active ROS production, glycolytic adenosine triphosphate (ATP) production, and cytoskeleton rearrangement (3, 18, 19). It is worth noting that ROS-independent NET formation has also been described (20). Recently, NET formation and neutrophil degranulation were demonstrated to be limited by the upregulation of the RHO GTPase RHOH upon neutrophil activation, resulting in the reduced transport of granules and mitochondria along actin filaments (21). Furthermore, NET formation was reported to occur independently of autophagy (22), gasdermin D, and pyroptotic cell death (17).

In this study, we aimed to define the role of the SNARE proteins syntaxin-4 and SNAP23 in the process of extracellular trap formation in both human circulating neutrophils and eosinophils *in vitro*. To this end, the interaction of syntaxin-4 and SNAP23 with their SNARE protein partners was blocked with chemically synthesized TAT-fusion peptides (TAT-syntaxin-4 and TAT-SNAP23, respectively) containing the corresponding conserved SNARE domain fused to the human immunodeficiency virus (HIV) transactivator of transcription (TAT) sequence, as previously described (7, 23). Our findings indicate that syntaxin-4 and SNAP23 are involved in ROS production as well as neutrophil azurophilic and specific granules exocytosis. However, the release of SVs and mtDNA was not impaired following pretreatment with the TAT-fusion peptides. These data suggest that the molecular pathways involved in neutrophil degranulation and mtDNA release are at least partially distinct. Furthermore, even though syntaxin-4 and SNAP23 are expressed by human eosinophils, we demonstrate that neither degranulation nor the release of mtDNA relies on these SNARE proteins subsequent to granulocyte-macrophage colony-stimulating factor (GM-CSF) priming and complement component 5a (C5a) stimulation.

## 2 Materials and methods

### 2.1 Reagents

Pancoll Human was purchased from PAN-Biotech GmbH (Aidenbach, Germany). Fetal calf serum (FCS) was obtained from GE Healthcare Life Science (Freiburg, Germany). GM-CSF was supplied by Novartis Pharma (Nuremberg, Germany). Human recombinant C5a was purchased from Hycult Biotech (Uden, The Netherlands). Phorbol 12-myristate 13-acetate (PMA), and diphenyleneiodonium chloride (DPI) were purchased from Calbiochem (distributed by Sigma-Aldrich (Buchs, Switzerland)). Dimethylsulfoxide (DMSO), p-nitrophenyl-2-acetamido-2-deoxy-α-D-glucopyranoside, *N*-formylmethionyl-leucyl-phenylalanine (fMLF), phosphate-buffered saline (PBS), bovine serum albumin (BSA), saponin, potassium bicarbonate (KHCO_3_), Triton X-100, and dihydrorhodamine 123 (DHR123) were from Sigma-Aldrich (Buchs, Switzerland). Prolong Gold mounting media, Hoechst 33342, the Quant-iT PicoGreen dsDNA assay kit, propidium iodide (PI), ethylenediaminetetraacetic acid (EDTA, pH 8.0), RPMI-1640/GlutaMAX medium were obtained from Thermo Fisher Scientific (distributed by LuBioScience GmbH, Lucerne, Switzerland). X-VIVO 15 medium without phenol red and antibiotics was purchased from Lonza (Walkersville, MD, USA). Proteinase K was from Roche Diagnostics (Rotkreuz, Switzerland), and deoxyribonuclease I (DNase I) from Worthington Biochemical Corporation (Lakewood, NJ, USA). Mouse FITC-conjugated anti-human myeloperoxidase (MPO, clone MPO-7) was obtained from Agilent Dako (Santa Clara, USA). APC-conjugated anti-human CD63 (clone H5C6), FITC-conjugated anti-human CD66b (clone G10F5), and PE-conjugated anti-human CD35 (clone E11) were from BioLegend (London, UK). Polyvalent human IgG was a gift from CSL Behring (Bern, Switzerland). Normal goat serum was from Santa Cruz Biotechnology, Inc (Heidelberg, Germany), and ChromPure human IgG from Jackson ImmunoResearch Laboratories Inc (Philadelphia, PA, USA). German glass coverslips (#1 thickness, 12-mm diameter) were obtained from Hecht-Assistant (Altnau, Switzerland). Black glass-bottom 96-well plates were from Greiner Bio-One GmbH (Frickenhausen, Germany). Ammonium chloride (NH_4_Cl) and Hemacolor Rapid staining kit were purchased from Merck Millipore (Darmstadt, Germany).

### 2.2 TAT-syntaxin-4, TAT-SNAP23 and TAT-control peptides

The sequences of TAT-syntaxin-4 and TAT-SNAP23 peptides were adapted from Uriarte et al. by fusing the t-SNARE coiled-coil homology domain of the respective SNARE proteins syntaxin-4 and SNAP23 to the TAT sequence (7) (TAT-syntaxin-4: YGRKKRRQRR RRHSEIQQLE RSIRELHDIF TFLATEVEMQ GEMINRIEKN IL; TAT-SNAP23: YGRKKRRQRR RHQITDESLE STRRILGLAI ESQDAGIKTI TMLDEQKEQL NRIEEGLDQI NKDMRETEKT LTEL). Both peptides were synthesized at a purity level of more than 95% (GL Biochem, Shanghai, China) and dissolved in 100% DMSO.

TAT-control peptide (FITC-ahx-GRKKRRQRRR PPQ) was kindly offered by Prof. Thomas Kaufmann (Institute of Pharmacology, University of Bern). The peptide was synthesized at a purity level greater than 95% (GL Biochem Shanghai, China) and dissolved in ddH_2_O.

### 2.3 Purification of human blood neutrophils and eosinophils

Human blood neutrophils and eosinophils were purified from peripheral human blood as previously described (11, 19). In brief, white blood cells were layered on Pancoll Human (density of 1.077 g/mL, PAN-Biotech, Aidenbach, Germany) and separated using density-gradient centrifugation (20 min, 800 x *g*, room temperature (RT)). The remaining erythrocytes in the granulocyte fraction were lysed with lysis buffer containing 155 mM NH_4_Cl and 10 mM KHCO_3_. For neutrophil experiments, only resulting cell populations with ≥ 95% purity assessed by an automated hematology analyzer (Sysmex Digitana, Horgen, Switzerland) were considered. For eosinophil experiments, an EasySep Human Eosinophil Isolation Kit (StemCell Technologies, Cologne, Germany) was used to isolate eosinophils from the granulocyte fraction by negative selection. Eosinophil purity (≥ 97%) was assessed using Hemacolor Rapid staining kit (Merck Millipore, Darmstadt, Germany) and light microscopic analysis.

Written, informed consent was obtained from all blood donors. The Ethics Committee of the Canton of Bern approved this study.

### 2.4 Activation of neutrophils and eosinophils

Isolated human neutrophils and eosinophils were pretreated with 5 µg/mL TAT-syntaxin-4, TAT-SNAP23, and/or TAT-control or 50 µM DPI in selected experiments at 37°C for 30 min. Subsequently, cells were primed with 25 ng/mL GM-CSF for 20 min and stimulated with 10 nM C5a or 1 µM fMLF at 37°C for the indicated time points. Unprimed cells were also stimulated with 25 nM PMA at 37°C for the indicated times.

### 2.5 Degranulation assays

#### 2.5.1 Surface expression of surrogate markers

Degranulation was assessed by the increase in surface expression of surrogate markers as previously described (11, 18). Briefly, freshly purified human neutrophils and eosinophils (0.5 x 10^6^ cells/200 µL) were resuspended in X-VIVO 15 medium and stimulated as described above. Neutrophil degranulation of azurophilic granules, specific granules, and SVs were determined using the following mAbs: APC-conjugated anti-human CD63 (clone H5C6; Cat # 353008; BioLegend, London, UK; 1:50 dilution), FITC-conjugated anti-human CD66b (clone G10F5; Cat # 305104; BioLegend, London, UK; 1:50 dilution) and PE-conjugated anti-human CD35 (clone E11; Cat # 333406; BioLegend, London, UK; 1:50 dilution).

Eosinophil degranulation of specific granules was determined using APC-conjugated anti-human CD63 antibody (clone H5C6; Cat # 353008; BioLegend, London, UK; 1:50 dilution).

Cells were acquired by flow cytometry (FACSVerse, BD Biosciences) and analyzed using FlowJo software (Tree Star, Ashland, OR, USA).

#### 2.5.2 Release of matrix metalloproteinase-9

Degranulation of the neutrophil gelatinase granule protein matrix metalloproteinase (MMP)-9 was assessed by ELISA (BioLegend) as previously described (7, 18). Briefly, freshly purified human neutrophils (0.5 x 10^6^ cells/200 µL) were resuspended in X-VIVO 15 medium and stimulated as described above. Supernatants were collected following centrifugation (5 min, 1400 rpm, 4°C) and the release of MMP-9 was measured according to the manufacturer’s protocol. The absorbance was measured at 450 nm with wavelength correction at 570 nm using a SpectraMax M2 plate reader (Molecular Devices, Biberach an der Riss, Germany).

#### 2.5.3 Release of *N*-acetyl-β-glucosaminidase

The colorimetric detection of *N*-acetyl-β-glucosaminidase (NAG) in the supernatants of stimulated cells was adapted from previous reports (18, 19, 24). Briefly, freshly purified human neutrophils (2 x 10^6^ cells/200 µL) were resuspended in X-VIVO 15 medium and stimulated as described above. Supernatants were collected following centrifugation (20 min, 2000 rpm, 4°C). Cell pellets were lysed with 0.12% Triton X-100 for 10 min at RT, followed by a second centrifugation step (20 min, 2000 rpm, 4°C) to remove cell debris. The substrate solution (5 mM p-nitrophenyl-2-acetamido-2-deoxy-α-D-glucopyranoside in 25 mM sodium citrate, pH 4.5) was added to the cell lysate and supernatant for 1h at 37 °C. The absorbance was measured at 410 nm using a SpectraMax M2 plate reader (Molecular Devices). The amount of NAG released in the supernatant of neutrophils was expressed as a percentage of the total NAG.

### 2.6 Reactive oxygen species measurements

The measurement of ROS production was adapted from a previous study (18). Briefly, freshly purified human neutrophils (0.25 x 10^6^ cells/250 µL) and eosinophils (0.5 x 10^6^ cells/250 µL) were resuspended in RPMI-1640 supplemented with 5% FCS and stimulated as described above. DHR123 was added to the cells with a final concentration of 1 µM at the end of priming with GM-CSF. Subsequently, the cells were activated and 100 µL of cell suspension was added to a black, glass-bottom 96-well plate in duplicates. The ROS activity of the samples was immediately measured every 5 min over the time period of 1h using a SpectraMax M2 plate reader (Molecular Devices) equipped with a 37°C incubator.

### 2.7 Analyses of extracellular DNA trap staining

Staining of extracellular DNA traps was performed as previously described (19). Briefly, freshly purified human neutrophils were resuspended (0.25 x 10^6^ cells/100 µL) in X-VIVO 15 medium, seeded on 12 mm glass coverslips, and stimulated as described above. Cells were fixed with 4% paraformaldehyde for 10 min, washed with PBS, and permeabilized with 0.05% saponin in PBS for 3 min at RT. Subsequently, the cells were washed with 0.005% saponin in PBS and stained in presence of 0.01% saponin. Cells were blocked in blocking buffer containing 1% ChromPure human IgG for 30 min at RT. Direct immunofluorescence staining was performed by using monoclonal mouse FITC-conjugated anti-human MPO (1:100, clone MPO-7, Cat #F0714, Agilent Dako, Santa Clara, USA) for 1h at RT. Cells were washed in PBS, stained with 10 µg/mL PI for 10 min at RT, and mounted with Prolong Gold Antifade mounting medium.

Images were acquired by confocal laser scanning microscopy (LSM 800 with Airyscan, Carl Zeiss Micro Imaging, Jena, Germany) using a Plan-Apochromat 63x/1.40 Oil DIC objective and analyzed with Imaris software (Bitplane AG, Zurich, Switzerland). Ten representative pictures from each condition were subjected to analysis to measure mean fluorescence intensity (MFI) of MPO (green channel) intracellularly and within extracellular traps using the surface module of Imaris software.

### 2.8 Quantification of released dsDNA in culture supernatants

Freshly purified human neutrophils and eosinophils were resuspended (1 x 10^6^ cells/500 µL) in X-VIVO 15 medium and stimulated as described above.

In eosinophils, in the last 2 min of activation time, 2.5 U/mL DNase I and 0.2 mg/mL proteinase K were added, as previously described (11). In neutrophils, 2.5 U/mL DNase I was added in the last 10 min of activation time, similar to a previous study (19).

Subsequently, 2.5 mM EDTA (pH 8.0) was added to the cells to stop reactions. Cells were pelleted at 1’400 rpm for neutrophils and 13’000 rpm for eosinophils for 5 min. 100 µL supernatant was transferred to black, glass-bottom 96-well plates in duplicates. The fluorescent activity of PicoGreen dye bound to dsDNA was excited at 480 nm and fluorescence emission intensity was measured at 520 nm using a spectrofluorometer (SpectraMax M2, Molecular Devices) according to the instructions described in the Quant-iT™ PicoGreen™ assay kit.

### 2.9 TAT uptake staining

Freshly purified human neutrophils and eosinophils were resuspended (0.25 x 10^6^ cells/200 µL) in X-VIVO 15 medium and incubated with 5 µg/mL FITC-conjugated TAT-control peptide for the indicated time points at 37°C. Subsequently, cells were washed, seeded on 12 mm glass coverslips, and fixed with 4% paraformaldehyde for 10 min. Cells were washed with PBS, stained with Hoechst 33342 (1 µg/mL) for 10 min at RT, and mounted with Prolong Gold Antifade mounting medium. Cells were acquired by confocal laser scanning microscopy (LSM 800, Carl Zeiss Micro Imaging) using a Plan-Apochromat 63x/1.40 Oil DIC objective and analyzed with Imaris software (Bitplane AG).

### 2.10 TAT uptake assay

Freshly purified human neutrophils and eosinophils were resuspended (0.5 x 10^6^ cells/200 µL) in X-VIVO 15 medium and incubated with 5 µg/mL FITC-conjugated TAT-control peptide at 37°C for the indicated time points. Subsequently, cells were washed and acquired by flow cytometry (FACSVerse, BD Biosciences). The MFI of FITC was analyzed using FlowJo software (Tree Star).

### 2.11 Cell viability assay

Cell viability was measured as previously described (17, 22). Briefly, freshly purified human neutrophils and eosinophils were resuspended (0.1 x 10^6^ cells/100 µL) in RPMI-1640 supplemented with 2% FCS and stimulated as described above. Cell death was assessed by the uptake of 10 µg/mL PI. Cells were acquired by flow cytometry (FACSVerse, BD Biosciences) and analyzed using FlowJo software (Tree Star).

### 2.12 Statistical analysis

GraphPad Prism 8 software (GraphPad Software Inc., La Jolla, CA, USA) was used for the analysis of the data. Data are presented as mean values ± SEM. To compare groups, one-way ANOVA with Tukey’s multiple comparisons test was used. Two-way ANOVA with Dunnett’s multiple comparisons test was applied to compare groups at different time points. *p* values ≤ 0.05 were considered statistically significant.

## 3 Results

### 3.1 Syntaxin-4 and SNAP23 are involved in neutrophil degranulation and ROS production

**Figure 1:**
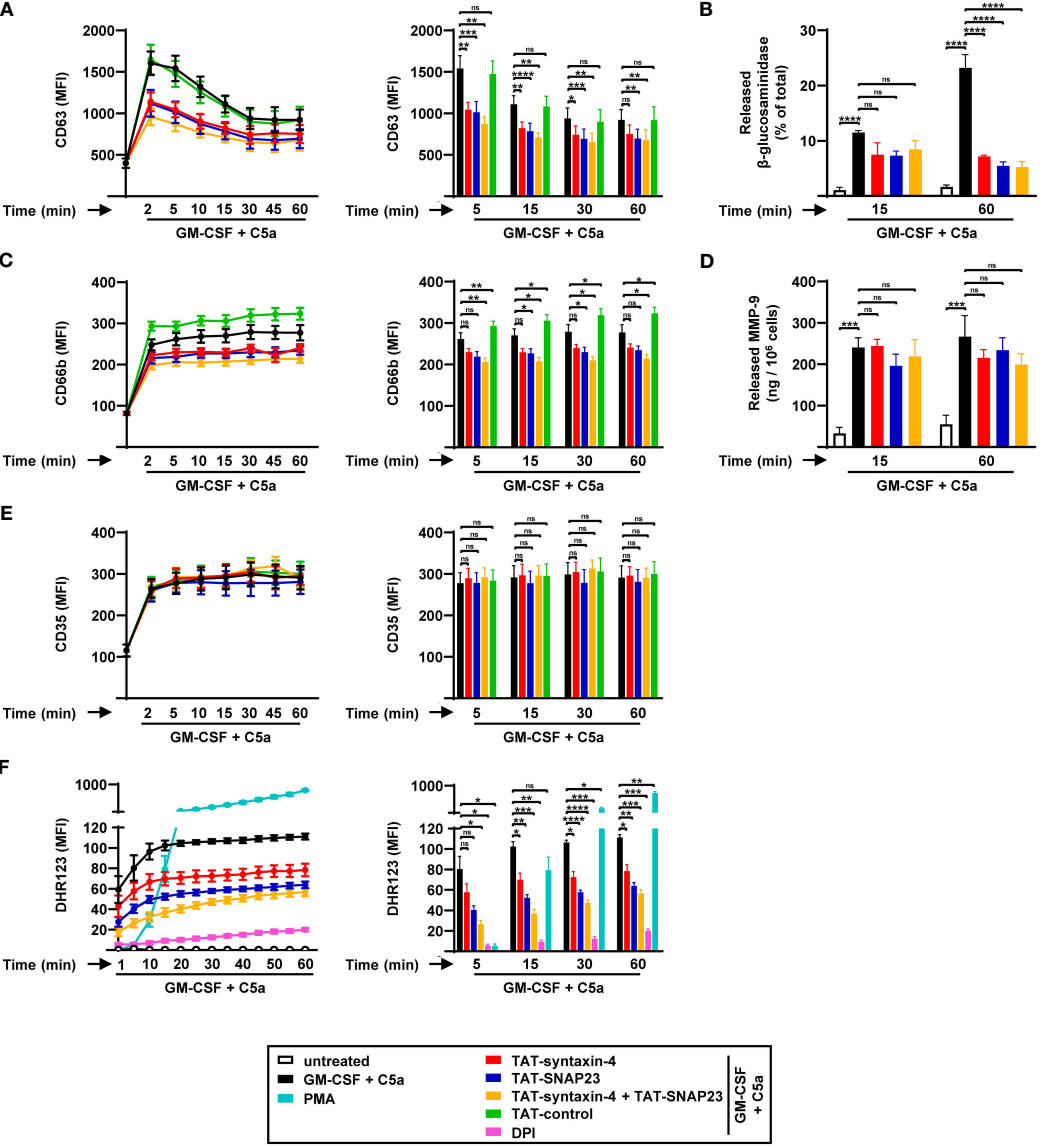
Time-dependent degranulation and ROS production by human circulating neutrophils stimulated with GM-CSF/C5a. **(A-E)** Degranulation assays. Isolated human neutrophils were pretreated with TAT-syntaxin-4, TAT-SNAP23, and/or TAT-control-FITC for 30 min, primed with GM-CSF for 20 min, and stimulated with C5a at the indicated time points. Neutrophil degranulation was assessed by CD63 surface expression (*n* = 7) **(A)** and the release of *N*-acetyl-β-glucosaminidase (*n* = 3) **(B)** for azurophilic granules, CD66b surface expression for specific granules (*n* = 7) **(C)**, the release of MMP-9 for gelatinase granules (*n* = 3) **(D)**, and CD35 surface expression for secretory vesicles (*n* = 7) **(E)**. **(A, C, E)** *Left*: Neutrophil degranulation kinetics following indicated treatments. *Right*: Bar plots representing the indicated time points of the kinetic curve with the corresponding statistical significances. **(F)** ROS production. Isolated human neutrophils were pretreated with TAT-syntaxin-4 and/or TAT-SNAP23 for 30 min, primed with GM-CSF for 20 min, and stimulated with C5a in a time-dependent manner (2 to 60 min). Unprimed neutrophils were activated with PMA. DPI was used as a negative control. ROS production was assessed by measuring DHR123 fluorescence with a spectrofluorometer (*n* = 4). *Left*: Neutrophil ROS production kinetics following indicated treatments. *Right*: Bar plots representing the indicated time points of the kinetic curve with the corresponding statistical significances. Values are means ± SEM. ns, not significant; *p < 0.05; **p < 0.01; ***p < 0.001; ****p < 0.0001.

Syntaxin-4 and SNAP23 were previously reported to be involved in the exocytosis of tumor necrosis factor (TNF)-primed and fMLF-stimulated human neutrophils (7). In order to investigate the contribution of syntaxin-4 and SNAP23 on the kinetics of GM-CSF/C5a-induced neutrophil degranulation, we assessed the surface levels of surrogate markers for azurophilic granules (CD63), specific granules (CD66b), and SVs (CD35) by flow cytometry in a time-dependent manner (**Figures 1A, C, E**). Freshly isolated blood neutrophils were pretreated with or without SNARE peptides (5 µg/mL) for 30 min, and degranulation and ROS activity were measured in the presence and absence of GM-CSF/C5a, or PMA alone. Our findings revealed that CD63 surface expression reached its peak within 2 min of C5a activation followed by a gradual decline (**Figure 1A**), possibly due to its tightly regulated trafficking between cellular locations (25). In contrast, CD66b and CD35 surface levels remained stable after a strong upregulation in the first 2 min of stimulation (**Figures 1C, E**). The TAT-fusion peptides TAT-syntaxin-4 and TAT-SNAP23, both separately and in combination, resulted in reduced CD63 surface levels, particularly within the first 15 min of stimulation (**Figure 1A**). We confirmed the strong inhibition observed in the surface expression of CD63 following competitive inhibition of syntaxin-4 and SNAP23 by measuring the release of NAG, a protein found in the azurophilic granules (18, 19) (**Figure 1B**). After 60 min of stimulation, TAT-syntaxin-4 and TAT-SNAP23 considerably decreased the release of NAG, both individually and in combination (**Figure 1B**). Furthermore, we detected decreased surface expression of CD66b upon treatment with TAT-syntaxin-4 and TAT-SNAP23, with statistical significance achieved over a time period of 60 min only when both peptides were employed together (**Figure 1C**). A similar trend of inhibition was observed when assessing the release of MMP-9, a key component of the gelatinase granules (26), but to a lower extent (**Figure 1D**). In contrast, CD35 surface levels remained unaltered (**Figure 1E**). Taken together, these results indicate that syntaxin-4 and SNAP23 contribute to the release of azurophilic and specific granules in GM-CSF/C5a-stimulated human neutrophils, while they may also have some involvement in gelatinase granules. However, their impact on the release of SVs appears to be negligible.

A previous study demonstrated that a significant proportion of the membrane-bound nicotinamide adenine dinucleotide phosphate (NADPH) oxidase subunit gp91^phox^ is associated with granule membranes of resting human neutrophils (27). Furthermore, the process of granule exocytosis leads to the translocation of gp91^phox^ to the plasma membrane, facilitating the assembly of the NADPH oxidase, and subsequent production of ROS (28, 29). Given the involvement of syntaxin-4 and SNAP23 in neutrophil degranulation, we investigated their impact on ROS production in GM-CSF/C5a-activated human neutrophils in a time-dependent manner (**Figure 1F**). Human neutrophils demonstrated increased production of ROS over time, eventually reaching a plateau after 20 min of treatment (**Figure 1F**). We observed a significant reduction of ROS levels in human neutrophils pretreated with TAT-syntaxin-4 and TAT-SNAP23, both individually and in combination (**Figure 1F**).

To confirm that human neutrophils efficiently internalize the TAT-fusion peptides, we investigated the uptake of the TAT-control peptide by confocal microscopy (**Figure S1A**). Neutrophils isolated from human peripheral blood were treated with FITC-conjugated TAT-control peptide for 10 to 30 min, followed by staining with Hoechst 33342 to visualize the nucleus. Using confocal microscopy, we observed intracellular localization of the TAT-control peptide (green) as early as 10 min after treatment of neutrophils (**Figure S1A**). Additionally, we quantified the uptake of the TAT-control peptide in human neutrophils using flow cytometry (**Figure S1B**). We observed strong increase in FITC levels, confirming the efficient internalization of TAT-fusion peptides already after 10 min. Furthermore, the FITC levels of the TAT-control peptide were slightly increased after 30 min of treatment compared to 10 min, implying that the TAT-fusion peptides were not degraded in this time period. Collectively, our findings demonstrate the rapid and stable uptake of TAT-fusion peptides by human neutrophils.

### 3.2 Syntaxin-4 and SNAP23 are not required for dsDNA release during the process of NET formation

**Figure 2:**
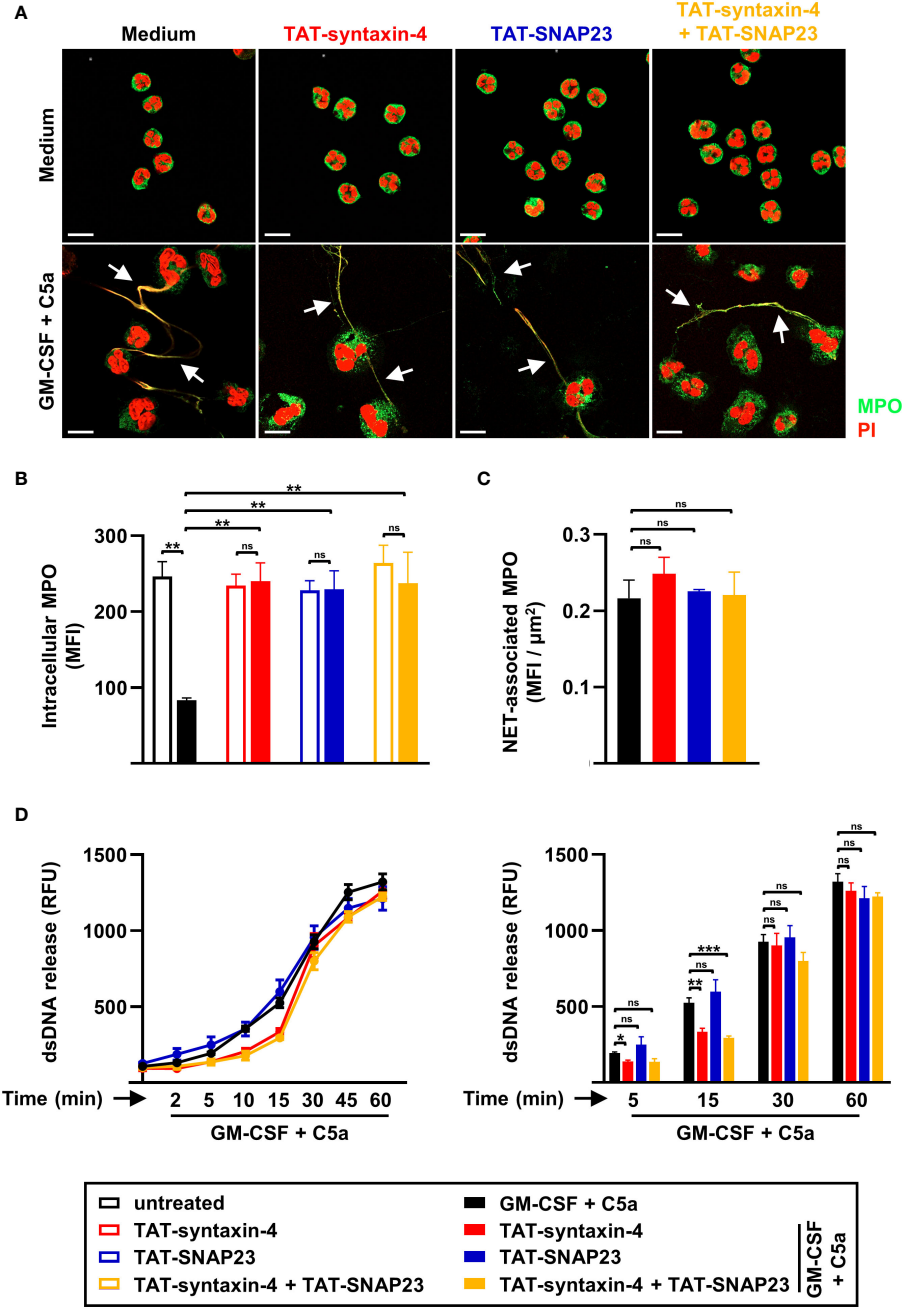
NET formation by circulating human neutrophils stimulated with GM-CSF/C5a. **(A)** Confocal microscopy. Isolated human neutrophils were pretreated with TAT-syntaxin-4, and/or TAT-SNAP23 for 30 min, primed with GM-CSF for 20 min, and stimulated with C5a for 60 min. Neutrophils were stained with propidium iodide (PI) (red) and monoclonal mouse anti-MPO-FITC antibody (green) (*n* = 3). Colocalizations of MPO within NETs are depicted by white arrows. Scale bar, 10 µm **(B, C)** Quantification of MPO mean fluorescence intensity (MFI). Quantification of remaining intracellular MPO (MFI) **(B)** and MPO within traps (MFI) **(C)** was performed using “Surfaces” analysis in Imaris software (*n* = 3). **(D)** dsDNA release assay. Isolated human neutrophils were pretreated with TAT-syntaxin-4, and/or TAT-SNAP23 for 30 min, primed with GM-CSF for 20 min, and stimulated with C5a in a time-dependent manner (2 to 60 min). Quantification of released dsDNA in supernatants of activated human neutrophils was assessed by measuring PicoGreen fluorescent dye with a spectrofluorometer (*n* ≥ 3). *Left*: Neutrophil dsDNA release kinetics following indicated treatments. *Right*: Bar plots representing the indicated time points of the kinetic curve with the corresponding statistical significances. Values are means ± SEM. ns, not significant; *p < 0.05; **p < 0.01; ***p < 0.001.

While neutrophils release toxic granule content by degranulation, they also employ extracellular traps as an active defense mechanism (8). However, unlike the release of granule proteins, the mechanism by which dsDNA is released remains largely unidentified. To evaluate the role of syntaxin-4 and SNAP23 in the release of dsDNA, we assessed NET formation using confocal microscopy and stained DNA with PI and the azurophilic granule protein MPO with anti-MPO antibody (**Figure 2A**). We observed that the capability of human neutrophils to form extracellular traps was not impaired upon competitive inhibition of syntaxin-4 and/or SNAP23 (**Figure 2A**). In accordance with degranulation assessed by upregulation of the surrogate marker CD63 (**Figure 1A**), TAT-syntaxin-4 and TAT-SNAP23 significantly inhibited the release of MPO in GM-CSF/C5a stimulated human neutrophils (**Figure 2B**). Interestingly, the amount of MPO found embedded within the dsDNA scaffold remained unaffected by TAT-syntaxin-4 and/or TAT-SNAP23 pretreatment (**Figure 2C**). Additionally, we assessed the release of dsDNA in the extracellular space from human neutrophils with the same time-dependent stimulation as for the degranulation kinetics (**Figure 2D**). We observed a gradual release of dsDNA over time, with levels rising continuously until reaching their peak after 45 to 60 min (**Figure 2D**). In agreement with the confocal microscopy data, neither of the TAT-fusion peptides demonstrated a significant overall impact on the release of dsDNA. It is worth to note that TAT-syntaxin-4, but not TAT-SNAP23, appears to partially delay dsDNA release from GM-CSF/C5a-stimulated neutrophils in the early time points (5 to 15 min) of stimulation, although the overall dsDNA levels after 60 min are the same for the different conditions (**Figure 2D**). These findings suggest a delayed response rather than complete prevention of dsDNA release in response to TAT-syntaxin-4. Moreover, we confirmed that neither the formation of extracellular traps nor the treatment with TAT-syntaxin-4 and/or TAT-SNAP23 led to differences in the viability of human neutrophils (**Figure S2A**). In summary, we demonstrate that while syntaxin-4 and SNAP23 affect the degranulation of azurophilic and specific granules, they do not significantly contribute to the formation of NETs from human neutrophils.

### 3.3 Syntaxin-4 and SNAP23 have similar effects on the effector functions of GM-CSF-primed neutrophils stimulated with fMLF

**Figure 3:**
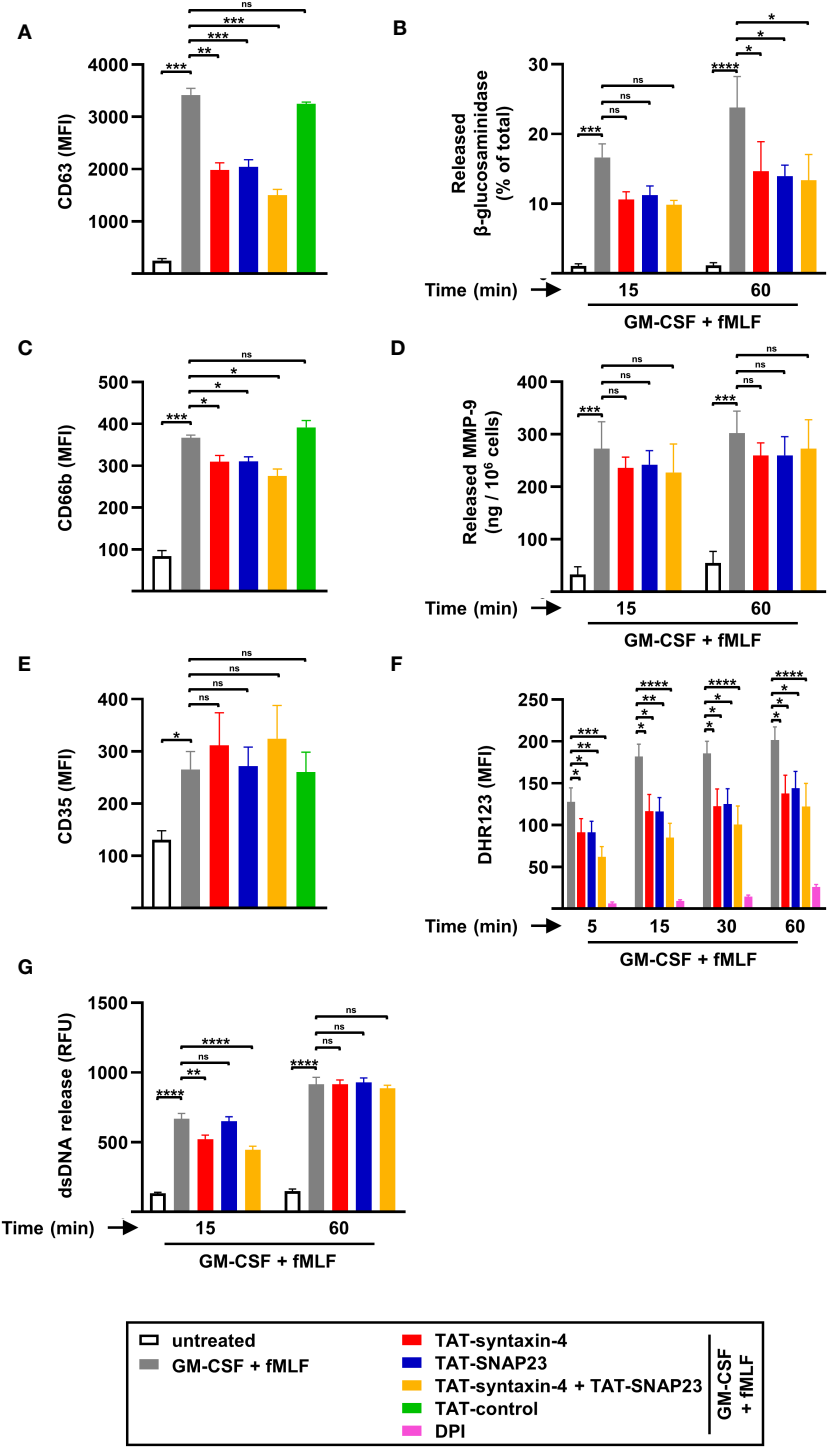
Degranulation, ROS production, and dsDNA released by circulating human neutrophils stimulated with GM-CSF/fMLF. **(A-E)** Degranulation assays. Isolated human neutrophils were pretreated with TAT-syntaxin-4, TAT-SNAP23, and/or TAT-control-FITC for 30 min, primed with GM-CSF for 20 min, and stimulated with fMLF for 5 min if not otherwise indicated. Neutrophil degranulation was assessed by CD63 surface expression (*n* = 4) **(A)** and the release of *N*-acetyl-β-glucosaminidase (*n* = 5) **(B)** for azurophilic granules, CD66b surface expression for specific granules (*n* = 4) **(C)**, the release of MMP-9 for gelatinase granules (*n* = 3) **(D)**, and CD35 surface expression for secretory vesicles (*n* = 4) **(E)**. **(F)** ROS production. Isolated human neutrophils were pretreated with TAT-syntaxin-4 and/or TAT-SNAP23 for 30 min, primed with GM-CSF for 20 min, and stimulated with fMLF at the indicated time points. DPI was used as a negative control. ROS production was assessed by measuring DHR123 fluorescence with a spectrofluorometer (*n* = 7). **(G)** dsDNA release assay. Isolated human neutrophils were pretreated with TAT-syntaxin-4, and/or TAT-SNAP23 for 30 min, primed with GM-CSF for 20 min, and stimulated with fMLF for 15 min and 60 min, respectively. Quantification of released dsDNA in supernatants of activated human neutrophils was assessed by measuring PicoGreen fluorescent dye with a spectrofluorometer (*n* = 5). Values are means ± SEM. ns, not significant; *p < 0.05; **p < 0.01; ***p < 0.001; ****p < 0.0001.

To further investigate the involvement of syntaxin-4 and SNAP23 in degranulation, ROS production, and dsDNA release, we adopted the same approach with additional agonists such as GM-CSF/fMLF (**Figure 3**) and PMA (**Figure S3**), both of which were reported to trigger NET formation (18, 19, 30). Based on our previous degranulation findings (**Figures 1A-E**), we investigated the surface levels of surrogate markers for azurophilic granules (CD63), specific granules (CD66b), and SVs (CD35) by flow cytometry in neutrophils stimulated with GM-CSF/fMLF or PMA for 5 min. Similar to GM-CSF/C5a, GM-CSF/fMLF-stimulated neutrophils demonstrated a strong increase in CD63 (**Figure 3A**) and CD66b (**Figure 3C**) surface expression after 5 min, which was partially inhibited following TAT-syntaxin-4 and/or TAT-SNAP23 pretreatment. In contrast, CD35 surface upregulation was not impaired upon competitive inhibition of syntaxin-4 and SNAP23 (**Figure 3E**). Furthermore, the release of NAG contained in azurophilic granules was also significantly decreased after 60 min of stimulation (**Figure 3B**), which is consistent with our flow cytometry data (**Figure 3A**), while the release of MMP-9 from gelatinase granules remained unaltered despite an apparent trend (**Figure 3D**). In addition, we noticed a similar pattern of ROS production in neutrophils stimulated with GM-CSF/fMLF (**Figure 3F**) compared to GM-CSF/C5a (**Figure 1F**). Similar observations were made regarding the release of dsDNA in the supernatant, which showed a slight delay after 15 min of stimulation in TAT-syntaxin-4-pretreated neutrophils that ultimately resulted in the same amount of released dsDNA after 60 min (**Figure 3G**). Interestingly, neutrophils stimulated with PMA demonstrated similar trends for degranulation (**Figures S3A-E**) and dsDNA release (**Figure S3G**), although to a lesser extent. Moreover, ROS production was not inhibited following TAT-syntaxin-4 and/or TAT-SNAP23 pretreatment, which contrasts with data obtained from neutrophils stimulated with GM-CSF/C5a (**Figure 1F**) or GM-CSF/fMLF (**Figure 3F**). Taken together, we demonstrate that syntaxin-4 and SNAP23 play a significant role in degranulation of azurophilic granules and secondary granules, as well as in ROS production, while they are not involved in the release of gelatinase granule, SVs, and dsDNA in neutrophils stimulated with GM-CSF/fMLF and GM-CSF/C5a.

### 3.4 Syntaxin-4 and SNAP23 do not participate in EET formation

**Figure 4:**
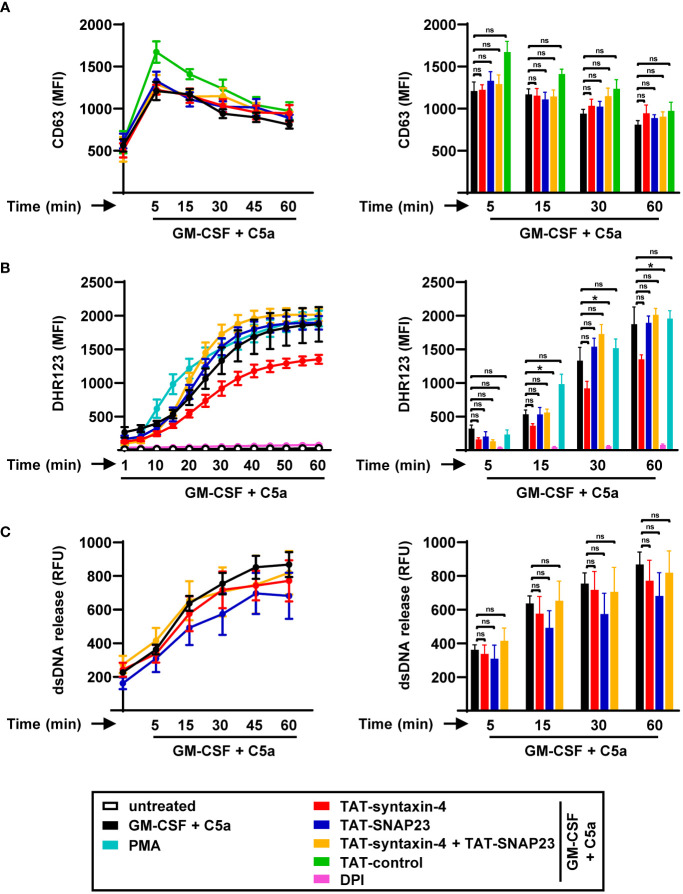
Time-dependent degranulation, ROS production, and EET formation by human circulating eosinophils stimulated with GM-CSF/C5a. **(A)** Degranulation assay. Isolated human eosinophils were pretreated with TAT-syntaxin-4, TAT-SNAP23, and/or TAT-control-FITC for 30 min, primed with GM-CSF for 20 min, and stimulated with C5a in a time-dependent manner (5 to 60 min). Eosinophil degranulation was assessed by CD63 surface expression using flow cytometry (*n* ≥ 3). *Left*: Eosinophil degranulation kinetics following indicated treatments. *Right*: Bar plots representing the indicated time points of the kinetic curve with the corresponding statistical significances. **(B)** ROS production. Isolated human eosinophils were pretreated with TAT-syntaxin-4, and/or TAT-SNAP23 for 30 min, primed with GM-CSF for 20 min, and stimulated with C5a in a time-dependent manner (5 to 60 min). Unprimed eosinophils were activated with PMA. DPI was used as a negative control. ROS production was assessed by measuring DHR123 fluorescence with a spectrofluorometer (*n* ≥ 3). *Left*: Eosinophil ROS production kinetics following indicated treatments. *Right*: Bar plots representing the indicated time points of the kinetic curve with the corresponding statistical significances. **(C)** dsDNA release assay. Isolated human eosinophils were pretreated with TAT-syntaxin-4, and/or TAT-SNAP23 for 30 min, primed with GM-CSF for 20 min, and stimulated with C5a in a time-dependent manner (5 to 60 min). Quantification of released dsDNA in supernatants of activated human eosinophils was assessed by measuring PicoGreen fluorescent dye with a spectrofluorometer (*n* ≥ 3). *Left*: Eosinophil dsDNA release kinetics following indicated treatments. *Right*: Bar plots representing the indicated time points of the kinetic curve with the corresponding statistical significances. Values are means ± SEM. ns, not significant; *p < 0.05.

SNARE proteins, which are widely recognized for their conserved function across all eukaryotic cells (31), are also present and expressed in eosinophils (32). Human eosinophils are also capable of degranulation and extracellular trap formation similar to human neutrophils (33). Therefore, we sought to explore the role of syntaxin-4 and SNAP23 in eosinophil functions (**Figure 4**). To assess eosinophil degranulation, we evaluated the time-dependent release of crystalloid granules using CD63 as a surrogate marker (**Figure 4A**), employing a similar approach as in human neutrophils (**Figure 1A**). We observed maximum release of CD63^+^ crystalloid granules within 5 min of C5a stimulation. In contrast to neutrophils, pretreatment with either TAT-syntaxin-4 and/or TAT-SNAP23 did not result in any inhibition of GM-CSF/C5a-induced degranulation in human eosinophils.

To demonstrate the capability of human eosinophils to efficiently take up the TAT-fusion peptides, we investigated the uptake of the TAT-control peptide using confocal microscopy (**Figure S1C**). Eosinophils were isolated from human peripheral blood, treated with the FITC-conjugated TAT-control peptide for 10 to 30 min, and stained with Hoechst 33342 for visualization of the nucleus. As in neutrophils (**Figure S1A**), we observed an intracellular localization of the TAT-control peptide (green) already 10 min after exposure to eosinophils (**Figure S1C**). Additionally, we assessed the intracellular content of TAT-control peptide in human eosinophils using flow cytometry (**Figure S1D**). Like in neutrophils, we observed strong uptake of the TAT-control peptide within 10 min and a further increase after 30 min, indicating that the TAT-fusion peptides are efficiently taken up by eosinophils and not degraded over time (**Figure S1D**). Collectively, these data provide evidence that TAT-fusion peptides are taken up both rapidly and stably by eosinophils confirming previous findings (34).

The generation of ROS plays a role in multiple processes of eosinophils, including the formation of eosinophil extracellular traps (EETs) (9), and other host defense mechanisms (35). Accordingly, we explored the role of syntaxin-4 and SNAP23 in the production of ROS in human eosinophils by assessing the kinetics of ROS production following GM-CSF/C5a and PMA stimulation in a time-dependent manner (**Figure 4B**). Over time, we observed a progressive rise in ROS production in human eosinophils. Consistent with our findings in eosinophil degranulation (**Figure 4A**), neither of the SNARE peptides exhibited relevant effects on the levels of ROS (**Figure 4B**). While TAT-SNAP23 alone and in combination with TAT-syntaxin-4 resulted in enhanced ROS production, TAT-syntaxin-4 individually lead to reduced levels of ROS, although the differences were not of statistical significance (**Figure 4B**).

To investigate whether syntaxin-4 and/or SNAP23 participate in the release of dsDNA within EET formation, we assessed the amount of extracellular DNA in the supernatant of human eosinophils following GM-CSF/C5a stimulation in a time-dependent manner (**Figure 4C**). Similar to human neutrophils (**Figure 2D**), we detected a gradual increase in the levels of released dsDNA from human eosinophils over time (**Figure 4C**). Moreover, the release of dsDNA was not significantly impaired by TAT-syntaxin-4 and/or TAT-SNAP23 treatment. Additionally, we demonstrated that the viability of human eosinophils remained unaffected by EET formation or treatment with TAT-syntaxin-4 and/or TAT-SNAP23 (**Figure S2B**). Collectively, our findings suggest that syntaxin-4 and SNAP23 do not contribute to degranulation, respiratory burst, and EET formation of human eosinophils stimulated with GM-CSF/C5a.

## 4 Discussion

Neutrophils play a crucial role in defending the host against various microorganisms (36). They employ distinct mechanisms such as degranulation, respiratory burst, phagocytosis, and NET formation as part of the initial defense line (16, 37). While SNARE proteins have been extensively studied for their involvement in membrane fusion events that occur during granule release (29, 31, 38), their contribution to NET formation has not been investigated yet. In line with previously published work (7), our study confirms that the introduction of TAT-fusion peptides derived from the SNARE proteins syntaxin-4 and SNAP23 results in decreased degranulation and diminished ROS levels in activated human neutrophils. Furthermore, we demonstrate that the release of mtDNA within the process of NET formation is not affected by the competitive inhibition of syntaxin-4 and SNAP23.

Previous studies reported that both syntaxin-4 and SNAP23 contribute to the release of specific granules, gelatinase granules, and secretory vesicles in fMLF- and PMA-stimulated neutrophils, whereas only syntaxin-4 participates in the degranulation of azurophilic granules (24, 29, 30). Interestingly, we demonstrate that competitive inhibition of both syntaxin-4 and SNAP23 in neutrophils results in diminished release of azurophilic and specific granules upon GM-CSF/C5a or GM-CSF/fMLF stimulation. While we observe a small reduction in the amount of released MMP-9 following competitive inhibition of syntaxin-4 and SNAP23, further investigations will be necessary to fully comprehend their involvement in the release of gelatinase granules in GM-CSF/C5a- or GM-CSF/fMLF-activated neutrophils. Additionally, it has been reported that inhibiting neutrophil degranulation also interferes with the production of ROS, indicating a mechanistic link between these two processes (23). In agreement with a previous study (29), we demonstrate reduced levels of ROS upon competitive inhibition of syntaxin-4 and SNAP23 in human neutrophils. In contrast, syntaxin-4 and SNAP23 seem to be involved to a lesser extent in degranulation but not in ROS production of neutrophils activated by PMA, emphasizing that distinct stimuli may ultimately dictate the involvement of different SNARE proteins. The differences observed in the inhibition of distinct granule types with previous publications may be attributed to variations in the preparation of TAT-fusion peptides.

In the context of NETs, granule proteins are found in association with mtDNA in the extracellular space (8). The precise molecular mechanism of how mtDNA is released during NET formation remains largely unknown. Given the reliance of NET formation on ROS production and degranulation, we hypothesized that SNARE proteins may play a role in the release of mtDNA. Surprisingly, we demonstrated no involvement of syntaxin-4 and SNAP23 in the release of mtDNA within the process of NET formation. It is worth noting that the competitive inhibition of syntaxin-4 leads to a slight delay in the kinetics of mtDNA release in the first 15 min of C5a stimulation of GM-CSF-primed neutrophils. Importantly, a similar effect is observed with both fMLF and PMA stimulation, highlighting that the process of mtDNA release does not significantly depend on syntaxin-4 and SNAP23, regardless of the agonist employed. Furthermore, while total suppression of ROS production has been previously demonstrated to prevent NET formation (18), the partial ROS inhibition subsequent to TAT-syntaxin-4 and TAT-SNAP23 pretreatment had no effect on the release of mtDNA, indicating that low levels of ROS are sufficient for NET formation. Moreover, the amount of released MPO in the extracellular structure of NETs is comparable to stimulated neutrophils despite reduced degranulation of azurophilic granules upon competitive inhibition of syntaxin-4 and SNAP23. These findings emphasize that the release of mtDNA and granule content found within NETs doesn’t rely on the SNARE proteins syntaxin-4 and SNAP23. In addition, the comparison of neutrophil degranulation and mtDNA release kinetics suggests at least partially distinct release mechanisms for both processes, consistent with previous observations in both human and mouse eosinophils (11).

According to a previous study, syntaxin-4 and SNAP23 are expressed by eosinophils and may serve as cognate receptors for the vesicle-associated membrane protein (VAMP)-2 and consequently play a role in eosinophil degranulation (39). Our study suggests that these two SNARE proteins have no impact on degranulation, ROS production, and mtDNA release in GM-CSF/C5a-activated eosinophils. It is worth noting that eosinophils display four distinct degranulation patterns which may vary depending on the specific agonist (32). These patterns possibly involve diverse interaction partners, highlighting the significant variety of the underlying mechanisms in eosinophil degranulation. Importantly, the specific type of degranulation that occurs following GM-CSF/C5a stimulation has not yet been determined. Moreover, it is important to mention, that the membrane-bound subunit of the NADPH oxidase, gp91^phox^, is located primarily at the plasma membrane of eosinophils in contrast to neutrophils (40). As a result, the production of ROS primarily occurs in the extracellular environment and thus does not require the translocation of NADPH oxidase subunits, which may explain the independence of SNARE proteins in the process.

Taken together, we provide evidence that the release of mtDNA within extracellular trap formation in human neutrophils and eosinophils is independent of the SNARE proteins syntaxin-4 and SNAP23. As both these proteins are involved in the release of azurophilic and specific granules in neutrophils, our data suggest that the mechanisms underlying mtDNA release and neutrophil degranulation are at least partially distinct. Importantly, the amount of the granule protein MPO within traps remained unaffected despite strong inhibition of azurophilic granule release, implying a role for syntaxin-4 and SNAP23 in neutrophil degranulation but not in NET formation. Furthermore, as previously observed in human and mouse eosinophils (11), we demonstrate for the first time that the release of mtDNA occurs after and independently of neutrophil degranulation. These data improve our current knowledge on NET formation. However, the exact mechanism regulating the release of mtDNA in the formation of extracellular traps is still not completely understood and requires further investigation.

## Data availability statement

The raw data supporting the conclusions of this article will be made available by the authors, without undue reservation.

## Ethics statement

The studies involving humans were approved by Ethics Committee of Canton of Bern. The studies were conducted in accordance with the local legislation and institutional requirements. The participants provided their written informed consent to participate in this study.

## Author contributions

LG: Formal Analysis, Investigation, Methodology, Writing – original draft. TF: Formal Analysis, Investigation, Methodology, Writing – original draft. MM: Investigation, Writing – original draft. KM: Conceptualization, Writing – review & editing. SY: Conceptualization, Formal Analysis, Funding acquisition, Investigation, Methodology, Software, Supervision, Writing – original draft, Writing – review & editing. DS: Conceptualization, Investigation, Methodology, Supervision, Writing – review & editing. HS: Conceptualization, Funding acquisition, Investigation, Project administration, Resources, Supervision, Writing – review & editing.

## Glossary

**Table:**

ATP	adenosine triphosphate
BSA	bovine serum albumin
C5a	complement component 5a
DHR123	dihydrorhodamine 123
DMSO	dimethylsulfoxide
DNA	deoxyribonuclease acid
DNase I	deoxyribonuclease I
DPI	diphenyleneiodonium chloride
dsDNA	double-stranded DNA
EDTA	ethylenediaminetetraacetic acid
EET	eosinophil extracellular trap
FCS	fetal calf serum
fMLF	N-formyl-methionyl-leucyl-phenylalanine
GM-CSF	granulocyte-macrophage colony-stimulating factor
HIV	human immunodeficiency virus
MFI	mean fluorescence intensity
MMP	matrix metalloproteinase
MPO	myeloperoxidase
mtDNA	mitochondrial DNA
NADPH	nicotinamide adenine dinucleotide phosphate
NAG	*N*-acetyl-β-glucosaminidase
NET	neutrophil extracellular trap
PBS	phosphate-buffered saline
PI	propidium iodide
PMA	phorbol 12-myristate 13-acetate
ROS	reactive oxygen species
RT	room temperature
SNAP23	synaptosomal-associated protein 23
SNARE	soluble N-ethylmaleimide-sensitive factor activating protein receptor
SV	secretory vesicle
TAT	transactivator of transcription
TNF	tumor necrosis factor
VAMP	vesicle-associated membrane protein
WBC	white blood cell
