# Supplementary material for: Syntaxin-4 and SNAP23 are involved in neutrophil degranulation, but not in the release of mitochondrial DNA during NET formation
Source: Front Immunol. 2023 Oct 9;14:1272699. doi: 10.3389/fimmu.2023.1272699 (PMC10599146; doi:10.3389/fimmu.2023.1272699)
Supplement: Supplementary file 1 [file Presentation_1.pdf]

Supplementary Material

Syntaxin-4 and SNAP23 are involved in neutrophil degranulation, but not in the release of mitochondrial DNA during NET formation

Lea Gigon[1,†], Timothée Fettrelet[1,†], Marta Miholic[1,2], Kenneth R. McLeish[3], Shida Yousefi[1], Darko Stojkov[1], and Hans-Uwe Simon[1,4*]

[1] Institute of Pharmacology, University of Bern, Bern, Switzerland

[2] Faculty of Pharmacy, University of Ljubljana, 1000 Ljubljana, Slovenia

[3] Department of Medicine, University of Louisville School of Medicine, Louisville, Kentucky, USA

[4] Institute of Biochemistry, Brandenburg Medical School, Neuruppin, Germany

***Correspondence:** Hans-Uwe Simon, hans-uwe.simon@unibe.ch

[†] These two authors contributed equally to this work and share first authorship.

Supplementary Figure count: 3

1 Supplementary Figures

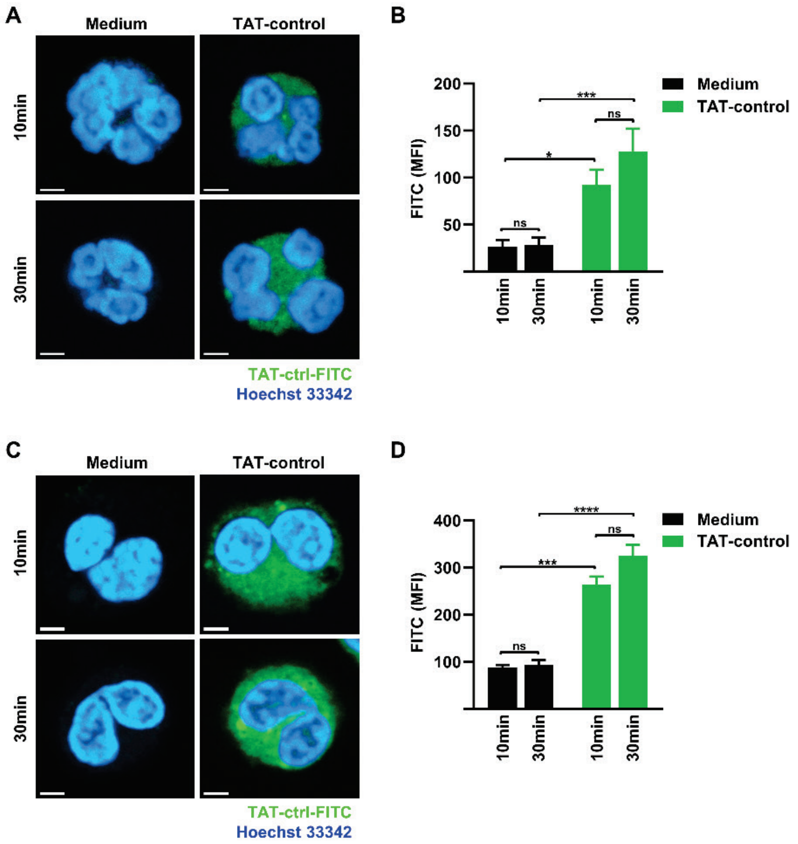

Supplementary Figure 1. Uptake of TAT-fusion peptide in human circulating neutrophils and eosinophils. (A, C) Confocal microscopy. Freshly isolated human neutrophils **(A)** and eosinophils **(C)** were pretreated with TAT-control-FITC (green) for 10 to 30 min, respectively. The nuclei were stained with Hoechst 33342 (blue). A representative image of TAT-control-FITC uptake is shown (n = 3). Scale bar, 2 µm. **(B, D)** Flow cytometry. Isolated human neutrophils (n = 6) **(B)** and eosinophils (n = 3) **(D)** were pretreated with TAT-control-FITC for 10 to 30 min, respectively. The uptake of TAT-control-FITC was assessed by FITC mean fluorescence intensity (MFI). Values are means ± SEM. ns not significant; * $p < 0.05$; *** p < 0.001; **** p < 0.0001.

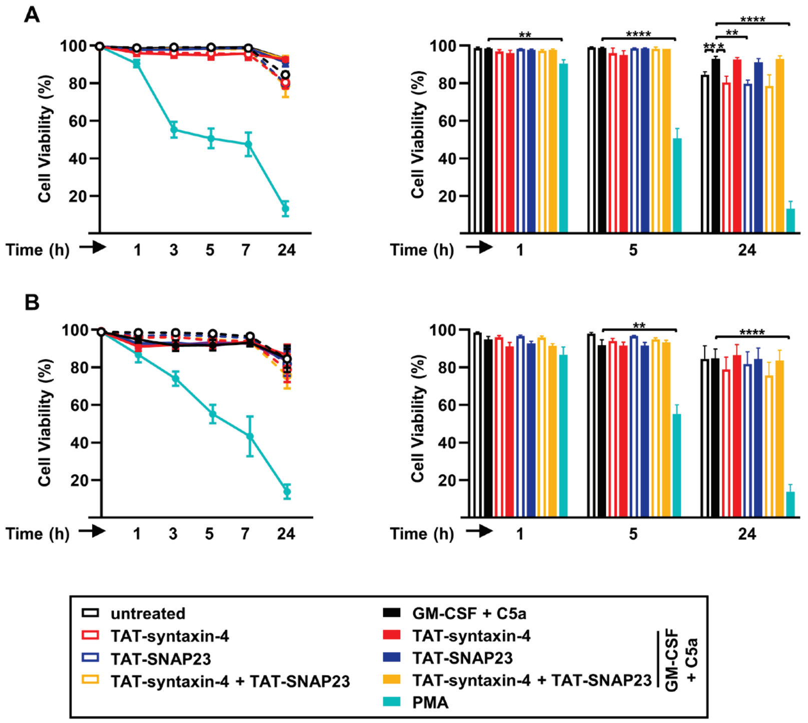

Supplementary Figure 2. Cell viability of TAT-syntaxin-4- and TAT-SNAP23-treated human neutrophils and eosinophils. (A, B) Viability assay. Isolated human neutrophils ($n \geq 3$) **(A)** and human eosinophils ($n \geq 4$) **(B)** were pretreated with TAT-syntaxin-4, and/or TAT-SNAP23 for 30 min, primed with GM-CSF for 20 min, and stimulated with C5a in a time-dependent manner (1 to 24h). Unprimed cells were activated with PMA. Cell viability was assessed by the uptake of propidium iodide (PI) using flow cytometry. *Left*: Neutrophil and eosinophil viability kinetics following indicated treatments. *Right*: Bar plots representing the indicated time points of the kinetic curve with the corresponding statistical significances. Values are means ± SEM. * $p < 0.05$; ** $p < 0.01$; **** $p < 0.0001$.

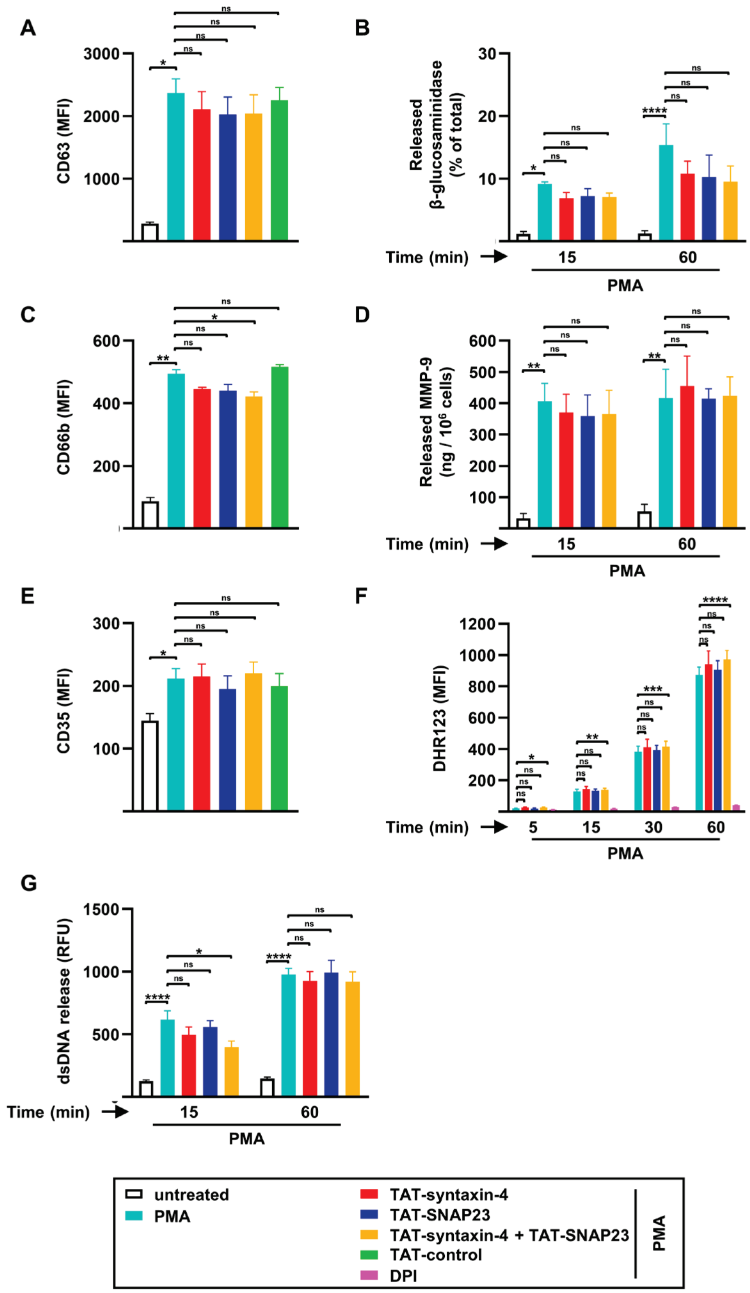

Supplementary Figure 3. Degranulation, ROS production, and NET formation by circulating human neutrophils stimulated with PMA. (A-E) Degranulation assays. Isolated human neutrophils were pretreated with TAT-syntaxin-4, TAT-SNAP23, and/or TAT-control-FITC for 30 min and stimulated with PMA for 5 min if not otherwise indicated. Neutrophil degranulation was assessed by CD63 surface expression ($n = 4$) **(A)** and the release of β-glucosaminidase ($n = 4$) **(B)** for azurophilic granules, CD66b surface expression for specific granules ($n = 4$) **(C)**, the release of MMP-9 for tertiary granules ($n = 3$) **(D)**, and CD35 surface expression for secretory vesicles ($n = 4$) **(E)**. **(F)** ROS production. Isolated human neutrophils were pretreated with TAT-syntaxin-4 and/or TAT-SNAP23 for 30 min and stimulated with PMA for the indicated time points. DPI was used as a negative control. ROS production was assessed by measuring DHR123 fluorescence with a spectrofluorometer ($n = 6$). **(G)** dsDNA release assay. Isolated human neutrophils were pretreated with TAT-syntaxin-4, and/or

TAT-SNAP23 for 30 min and stimulated with PMA for 15 min and 60 min, respectively. Quantification of released dsDNA in supernatants of activated human neutrophils was assessed by measuring PicoGreen fluorescent dye with a spectrofluorometer ($n = 5$). Values are means ± SEM. ns not significant; * $p < 0.05$; ** $p < 0.01$; *** $p < 0.001$; **** $p < 0.0001$.
